# Hyperintensities of middle frontal gyrus in patients with diabetic optic neuropathy: a dynamic amplitude of low-frequency fluctuation study

**DOI:** 10.18632/aging.203877

**Published:** 2022-02-04

**Authors:** Lin Yang, Ang Xiao, Qiu-Yu Li, Hui-Feng Zhong, Ting Su, Wen-Qing Shi, Ping Ying, Rong-Bin Liang, San-Hua Xu, Yi Shao, Qiong Zhou

**Affiliations:** 1 Department of Ophthalmology, Jiangxi Branch of National Clinical Research Center for Ocular Disease, The First Affiliated Hospital of Nanchang University, Nanchang 330006, Jiangxi, China; 2 Department of Intensive Care, The First Affiliated Hospital of Gannan Medical University, Ganzhou 341000, Jiangxi, China; 3 Department of Ophthalmology, Massachusetts Eye and Ear, Harvard Medical School, Boston, MA 02114, USA

**Keywords:** dynamic amplitude of low-frequency fluctuation, diabetic optic neuropathy, emotion function, functional network

## Abstract

Diabetic optic neuropathy (DON) is a diverse complication of diabetes and its pathogenesis has not been fully elucidated. The purpose of this study was to explore dynamic cerebral activity changes in DON patients using dynamic amplitude of low-frequency fluctuation (dALFF). In total, 22 DON patients and 22 healthy controls were enrolled. The dALFF approach was used in all participants to investigate dynamic intrinsic brain activity differences between the two groups. Compared with HCs, DON patients exhibited significantly increased dALFF variability in the right middle frontal gyrus (P < 0.01). Conversely, DON patients exhibited obviously decreased dALFF variability in the right precuneus (P < 0.01). We also found that there were significant negative correlations between HADS scores and dALFF values of the right middle frontal gyrus in the DON patients (r = -0.6404, P <0.01 for anxiety and r = -0.6346, P <0.01 for depression; HADS, Hospital Anxiety and Depression Scale). Abnormal variability of dALFF was observed in specific areas of the cerebrum in DON patients, which may contribute to distinguishing patients with DON from HCs and a better understanding of DON, hyperintensities of right middle frontal gyrus may be potential diagnostic marker for DON.

## INTRODUCTION

Diabetes mellitus (DM) is a common chronic disease and its prevalence continues to increase [1]. With progression of DM, neuropathy has become the most common symptomatic complication of diabetic patients [2]. Diabetic optic neuropathy (DON) is one of the major chronic complications of DM [3], and includes diabetic papillopathy, optic disc neovascularization, nonarthritic anterior ischemic optic neuropathy and optic atrophy [4]. The prevalence of DON in diabetic retinopathy (DR) patients is 38.4% [5]. Due to variation in the forms of DON, it is difficult to diagnose and it can therefore seriously threaten vision and affect the quality of life in diabetes patients, which underscores the need for an ophthalmologist’s evaluation. According to recent reports, hyperglycemia in diabetic patients decreases local tissue blood flow and this could affect the metabolism of the optic nerve [6]. The major mechanisms proposed to be involved in DON include activation of the polyol pathway, inflammatory response and oxidative stress [2, 7]. Furthermore, there is irreversible atrophy of the optic nerve as the disease progresses over time, which eventually leads to blindness.

The examination and diagnosis of optic neuropathy mainly relies on an ophthalmological fundus examination, such as fundus photography, visual evoked potentials (VEP) and fundus fluorescein angiography. However, these examinations may not be appropriate for all diabetic patients, especially in patients with heart failure, or liver failure, or renal failure, or drug allergies. In clinical practice, the prevalence of DON is usually underestimated [8], and there have been few studies on DON. This prompted us to adopt a new technique known as amplitude of low-frequency fluctuation (ALFF), to deepen our understanding of neural mechanism changes in DON patients. The ALFF examination is a fully automated, reliable, standardized and sensitive functional magnetic resonance imaging(MRI) technology that reflects the intensity of local spontaneous cerebrum activity and endogenous/background neurophysiological processes in the human cerebrum at rest [9], which has been shown to be a valuable parameter that reflects the intensity of spontaneous neural activity [10–12]. It is also a reliable biomarker for many neurological diseases and can be applied to the study of eye diseases, such as strabismus [13], amblyopia [14], glaucoma [15, 16], and retinal diseases [17–19].

**Figure 1:**
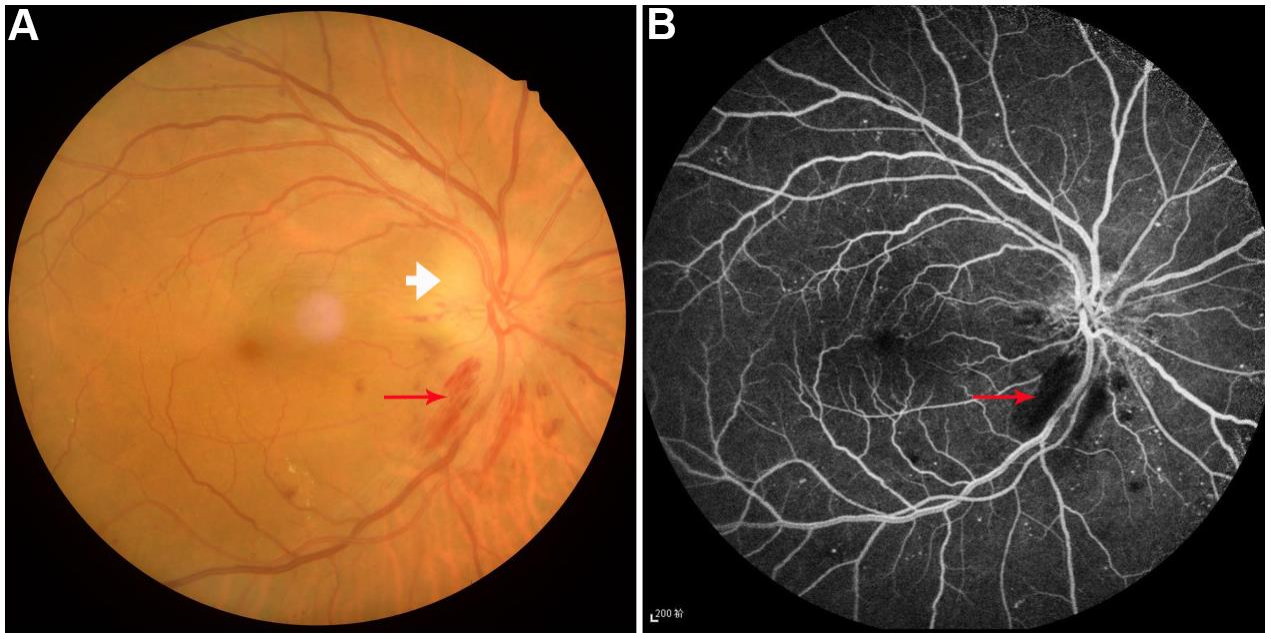
Example of diabetic optic neuropathy was performed on fundus camera (**A**) and fluorescence fundus angiography (**B**). Perioptic nerve hemorrhage (red arrow) and optic disc edema (white arrow) were observed.

However, the human cerebrum is the most complex and sophisticated organ in human body, and it produces continuous and rhythmic dynamic potential changes. It had been reported [20, 21] that the dynamic characteristics of cerebrum activity are related to various physiological functions, such as consciousness and cognition. Dynamic amplitude of low-frequency fluctuation (dALFF) is further measurement of the prolongation of ALFF, and it provides a new approach to explore ALFF on a time scale, which can allow dynamic study of local intrinsic cerebrum activity [22]. It has been applied to diagnosis and treatment of some diseases, such as depressive disorders [23] and schizophrenia [24, 25]. Currently, dALFF analysis has been extended and successfully applied to quantify and assess the dynamic cerebral activity changes in patients with generalized tonic-clonic seizures [26], poststroke aphasia [27], and diabetic retinopathy [28]. Due to the frontal, temporal and thalamus regions comprising a default mode network, they are involved in emotional, memory, and cognitive functions [29, 30]. Therefore, we hypothesized that there might be dynamic cerebral electrical activity changes in patients with DON (Figure 1), and connectivity changes in relevant cognition-related areas may result in depression and anxiety. Based on the above views, the purpose of this study was to determine whether altered dynamic spontaneous neural activity was observed in DON patients using the ALFF examinations for evaluation and analysis.

## RESULTS

### Clinical characteristics

In order to observed changes of dALFF values in DON patients, we recruited 22 DON patients and 22 health controls (HCs) who underwent the dALFF examination. There were no significant differences in sex, age, weight and handedness between the two groups (P>0.05). However, the binocular best corrected visual acuity of DON patients was significant worse than HCs (P < 0.05). Compared with HCs, DON patients showed delayed binocular latencies and lower amplitudes of VEP (P < 0.05). The mean course of DON patients was 56.76±5.26 days. Details are shown in Table 1.

**Table 1:** Clinical characteristics of participants in this study.

**Condition**	**DON**	**HCs**	***t***	***P* value**
Male/female	10/12	10/12	N/A	> 0.99
Age (years)	54.74 ± 5.98	53.02 ± 5.12	0.274	0.914
Weight (kg)	65.32 ± 7.52	62.12 ± 8.57	0.197	0.943
Handedness	22R	22R	N/A	> 0.99
Duration of DON (days)	56.76 ± 5.26	N/A	N/A	N/A
Best-corrected VA-left eye	0.35 ± 0.22	1.05 ± 0.25	-3.581	0.014
Best-corrected VA-right eye	0.26 ± 0.19	1.05 ± 0.15	-3.127	0.011
Latency (ms)-right of the VEP	121.01 ± 10.64	101.23 ± 5.42	3.291	0.002
Amplitude (μV)-right of the VEP	6.96 ± 2.15	13.29 ± 1.84	-8.021	0.003
Latency (ms)-left of the VEP	110.42 ± 7.48	100.76 ± 3.29	5.597	0.012
Amplitude (μV)-left of the VEP	10.26 ± 3.34	16.24 ± 2.65	-3.018	0.005

Note: Values are mean±SD.

Abbreviations: DON, Diabetic optic neuropathy; HCs, Healthy controls; VA, Visual acuity; N/A, Not applicable; VEP, Visual evoked potential.

χ2-test for sex and handedness(n).

Independent *t*-tests for other normally distributed continuous data.

### Variance differences of dALFF

**Figure 2:**
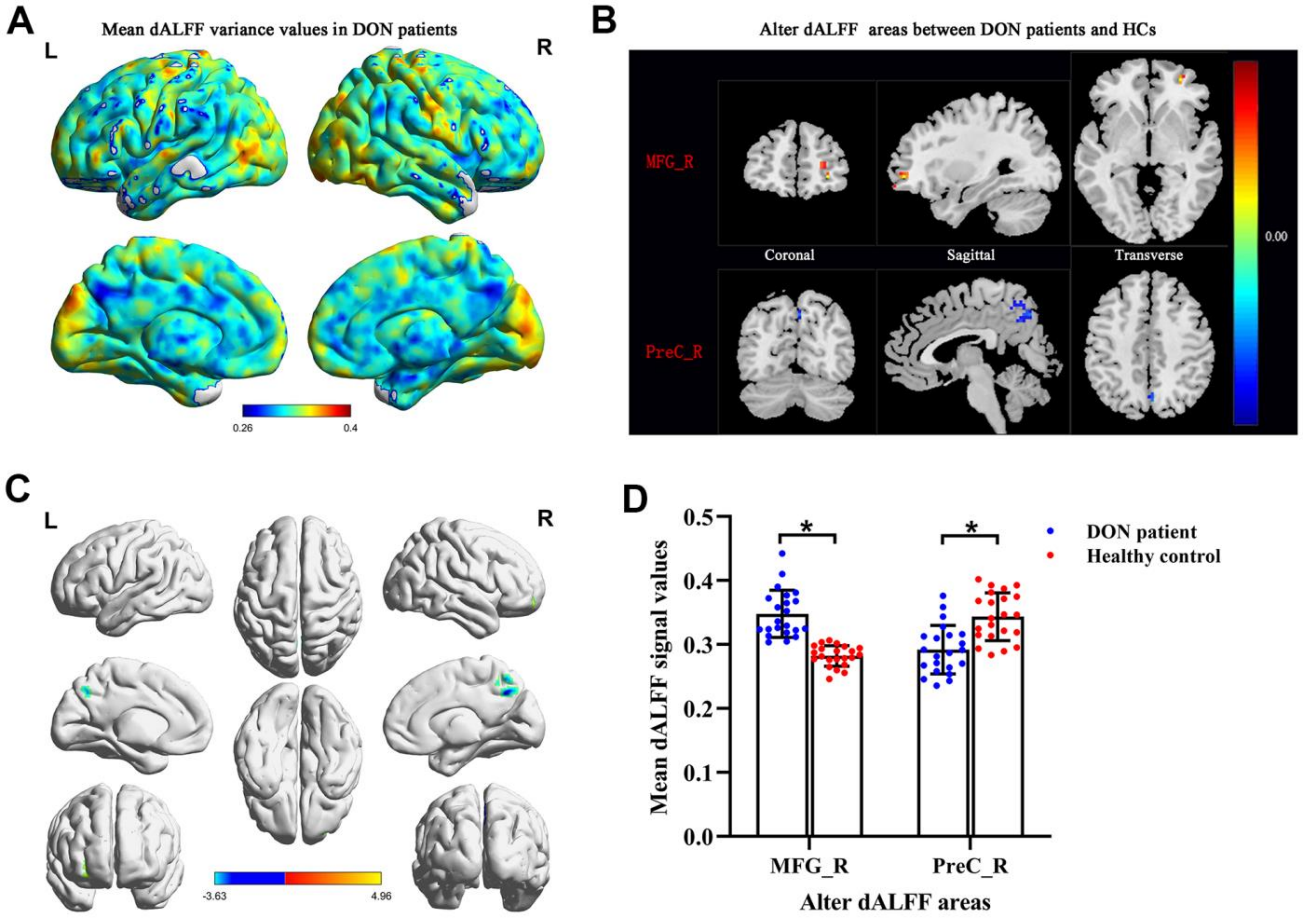
**Comparison of different dALFF values between the DON patients and HCs.** Spatial patterns of dALFF variance were observed in DON patients. The mean dALFF variance maps within DON patients (**A**). Compared with HCs, patients with DON showed increased MRG_R dALFF value and decreased PreC_R dALFF value. Representative distributions of them in the coronal, sagittal and transverse positions (**B**) and three-dimensional distributions (**C**). Two-sample t-test was used to compare dALFF values between two groups. The mean values of altered dALFF between the DON patients and HCs (**D**). * represented to P< 0.01. The warm color areas denote higher values, and the cool color areas denote lower values in two groups. Abbreviations: dALFF, dynamic amplitude of low-frequency fluctuation; DON, diabetic optic neuropathy; HCs, healthy controls; MFG, middle frontal gyrus; PreC, precuneus; R, right; L, left.

The spatial patterns of dALFF variability were determined in DON patients (Figure 2A) and HCs (data were not showed). Compared with HCs, DON patients exhibited increased dALFF variability in the right middle frontal gyrus (MFG_R) (Figure 2B, 2C and Table 2), and exhibited decreased dALFF variability in the right precuneus-(PreC_R) (Figure 2B, 2C and Table 2). The mean values of altered dALFF were shown in Figure 2D between the DON and HC groups. However, we found no significant abnormalities in other cerebrum regions.

**Table 2:** Brain regions with significantly different dALFF values between patients with DON and HCs.

**Brain area**	**BA**	**Voxel**	**MNI coordinates of peak voxel**			***t-value***
			**X**	**Y**	**Z**	
DON>HCs MFG_R	10	26	30	51	-3	4.9595
DON<HCs PreC_R	-	31	3	-69	45	-3.6307

Note: The statistical threshold was set at the voxel level with p < 0.01 for multiple comparisons using Gaussian random-field theory (two-tailed, voxel-level P < 0.01, GRF correction, cluster level P < 0.05).

Abbreviations: dALFF, dynamic amplitude of low-frequency fluctuation; DON, Diabetic optic neuropathy; HCs, healthy controls; BA, Brodmann area; R, right; MNI, Montreal Neurological Institute; MFG, middle frontal gyrus; PreC, precuneus; GRF, Gaussian random field.

### Receiver operating characteristic curve

**Figure 3:**
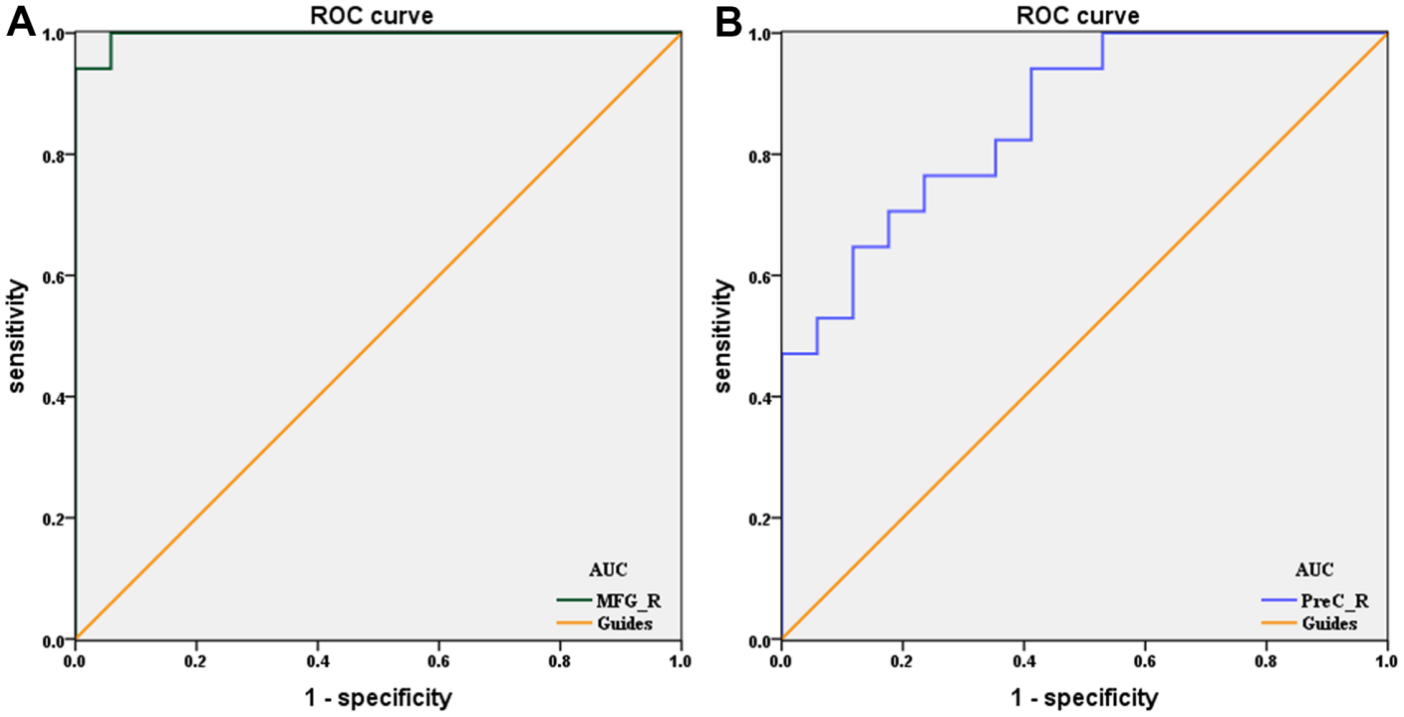
**ROC curve analysis of the mean dALFF values for altered brain regions.** (**A**) DON>HCs, the area under the ROC curve was 0.997 (P < 0.0001; 95% CI: 0.985–1.000) for MFG_R. (**B**) DON<HCs, the area under the ROC curve was 0.858 (P < 0.0001; 95% CI: 0.737–0.979) for PreC_R. Abbreviations: ROC, receiver operating characteristic; dALFF, dynamic amplitude of low-frequency fluctuation; DON, diabetic optic neuropathy; HCs, healthy controls; AUC, area under the curve; R, right; MFG, middle frontal gyrus; PreC, precuneus.

To verify whether differences in altered dALFF values would be applied to diagnostic biomarkers to differentiate the DON patients from HCs, receiver operating characteristic (ROC) curve analysis was used to analyze the mean altered dALFF values for specific cerebrum regions. The area under ROC curve (AUC) represents the diagnostic rate. Values of 0.5 to 0.7 are low accuracy, 0.7 to 0.9 are middle accuracy, and > 0.9 is high accuracy. The individual AUCs of altered dALFF values were as follows: DON > HCs, MFG_R (0.997, P < 0.0001, 95% CI: 0.985–1.000; Figure 3A); DON < HCs, PreC_R (0.858 P < 0.0001, 95% CI: 0.737–0.979; Figure 3B). These results indicated that dALFF values in altered brain areas may be helpful in diagnosing DON. Moreover, the ROC curves suggested that the MFG_R dALFF value had better clinic value than PreC_R.

### Correlation analysis

**Figure 4:**
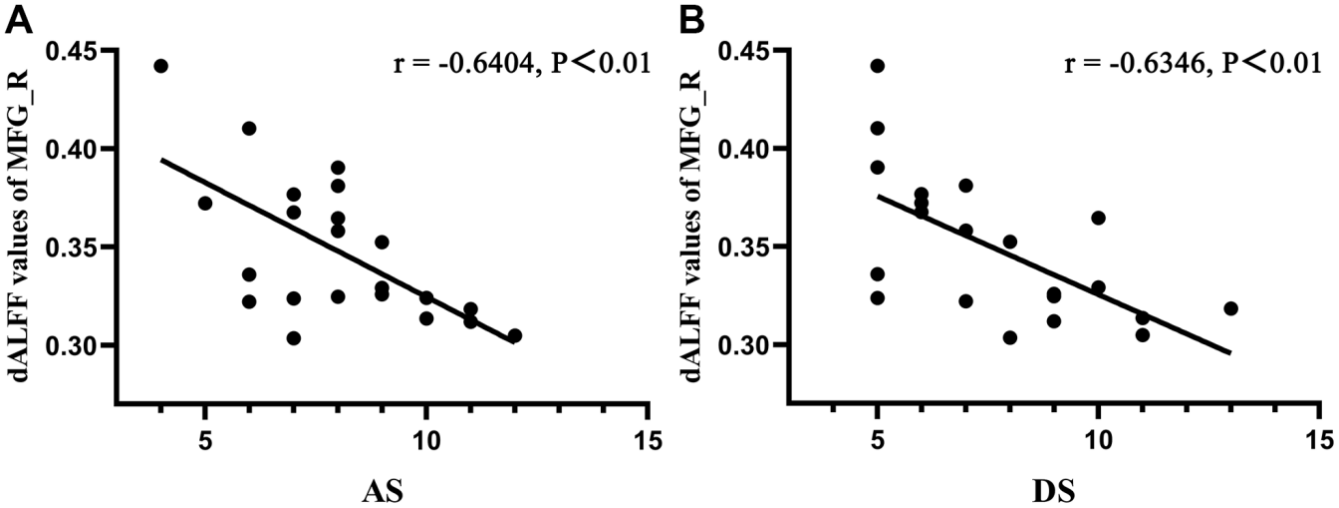
**Correlations between dALFF values of MFG_R and clinical behaviors.** A Pearson’s correlation analysis was performed to determine the linear relationship between significant dALFF values and HDS scores. (**A**) The anxiety scores showed a negative correlation with dALFF values of MFG_R (*r* = −0.6406, P < 0.01); (**B**) The depression scores showed a negative correlation with dALFF values of MFG_R (*r* = −0.6346, P < 0.01). Abbreviations: dALFF, dynamic amplitude of low-frequency fluctuation; MFG, middle frontal gyrus; R, right; AS, anxiety scores; DS, depression scores.

To determine the linear relationship between abnormal cerebrum regions and depression or anxiety, a Pearson’s correlation analysis was performed. Pearson's r can range from -1 to 1. An r of -1, 0 and 1 individually indicate a perfect negative, no, and a perfect positive linear relationship between variables. There were significant negative correlations between Hospital Anxiety and Depression Scale (HADS) scores and dALFF values of the MFG_R in DON patients (*r* = -0.6404, P <0.01 for anxiety and *r* = -0.6346, P <0.01 for depression; Figure 4). However, the relationship between dALFF values of PreC_R and HADS scores in patients with DON was not significant (P>0.05) (data were not showed).

## DISCUSSION

As we know, resting-state functional magnetic resonance imaging(rs-fMRI) signals are associated with resting state networks and used to assess individual cognitive, emotional and executive functions [31]. Alterations of cortical signals can be used to locate cortical functional areas and further study brain functions [32]. Recently, a lot of rs-fMRI methods were used to observe the brain activities in DON patients, like degree centrality [33], functional connection density [34], regional homogeneity [35] and voxel mirrored homotopic connectivity [36] methods (details in Table 3). This study is the first to use the dALFF method which was ALFF combined with sliding window to reveal the temporal variability of regional intrinsic cerebrum activities in DON patients. Our results indicated that DON patients exhibited increased dALFF values in the MFG_R and decreased dALFF values in the PreC_R compared with HCs. In addition, there were significant negative correlations between Hospital Anxiety and Depression Scale scores and dALFF values of the MFG_R in DON patients. These findings emphasize the importance of dynamic local brain activity in research.

**Table 3:** Rs-MRI method applied in DON patients in the current literatures.

**Author**	**Year**	**Method**	**Abnormal cerebrum regions**	
			**Decreased values**	**Increased values**
Xu et al. [33]	2020	DC	LFMO, RMFG/RGS	LTL
Chen et al. [34]	2021	FCD	longFCD: Bilateral Lingual, Lingual_R, Cingulum_Mid_L	lFCD: Cerebelum_8_L Cerebelum_Crus2_R Temporal_Inf_L Temporal_Sup_L
Guo et al. [35]	2021	ReHo	RMFG, LAC, SFG/ LFSO	-
Tan et al. [36]	2021	VMHC	RTI, LTI, RCM, LCM, RSM, LSM	-

Abbreviations: Rs-MRI, resting-state functional magnetic resonance imaging; DON, diabetes optic neuropathy; DC, [33]; LFMO, left frontal mid-orb; RMFG/RFS, right middle frontal gyrus /right frontal sup; LTL, left temporal lobe; FCD, functional connection density; R, right; L, left; ReHo, regional homogeneity; LAC, left anterior cingulate; SFG/ LFSO, superior frontal gyrus /left frontal superior orbital gyrus; VMHC, voxel mirrored homotopic connectivity; RTI, right temporal inf; LTI, left temporal inf; RCM, right cingulum mid; LCM, left cingulum mid; RSM, right supp motor; LSM, left supp motor.

In this study, DON patients showed worse binocular best corrected visual acuity because of optic disc inflammation or edema, resulting in delayed VEP latency and decreased amplitude. To our knowledge, VEP have been used to observe the transmission of light signals to a subcortical nucleus and the visual cortex and this illustrates the extent of the visual pathway [37]. Damage to somewhere of visual pathway can lead to visual impairment, which affects the normal lives and leads to negative emotions [38], cognitive impairment [39] and poor motor perception [40]. Previous studies had demonstrated that there were retinal ganglion cells loss or apoptosis [41, 42] and retinal nerve fiber layer thinner or loss in DR patients [43], but there were less than half of DR patients had optic neuropathy [5] and this neurodegeneration was an early event in the pathogenesis of DR [44]. Willemien et al. [45] observed the atrophy of retinal optic nerve and visual function impairment in patient with temporal lobe part resection, and revealed that it was caused by direct retrograde axial degeneration. Therefore, visual loss and cerebral cortex affect each other. According to delayed latencies and decreased amplitudes of VEP, damage of visual pathway was proved in DON patients. Several studies [46–48] also found the same results in DR or DON patients, but this method was nonspecific, the breakdown of visual information transmission in somewhere can cause to changes of VEP. We speculated that this change in DON patients was due to the disease itself, because we did not observe abnormal dALFF variability in occipital lobe.

**Figure 5:**
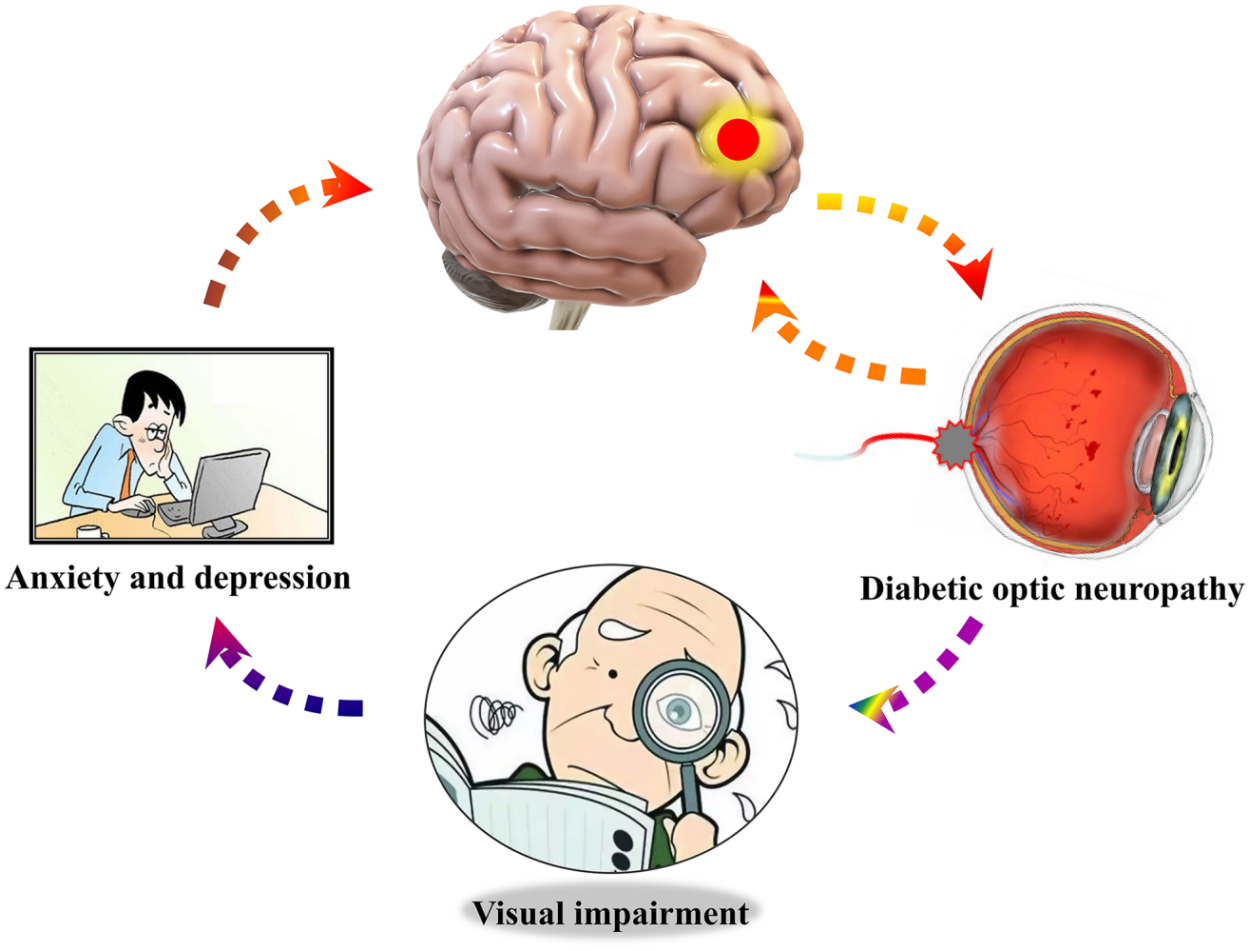
**Relationship between cerebrum dALFF values of right middle frontal gyrus and mood state.** Compared with HCs, dALFF values of right middle frontal gyrus were decreased in DON patients, which were more likely to undergo the anxiety and depression. Abbreviations: dALFF, dynamic amplitude of low-frequency fluctuation; HCs, healthy controls group; DON, diabetic optic neuropathy.

The optic nerve is part of the cerebrum and optic neuropathy may lead to changes of neural activity in some brain regions. Previous studies demonstrated that retinal neurovascular degeneration may be a potential biomarker for mild cognitive disorder and Alzheimer’s disease [49, 50]. Compared with HCs, we found increased dALFF variability in the right MFG of DON patients. The right MFG, as a part of the prefrontal cortex, is the combination of dorsal and ventral attention networks. It has the ability to interrupt attentional processes of endogenous stimuli and convert them to exogenous stimuli, as with a circuit-breaker [51]. Japee et al. [52] demonstrated that this function may be conducive to remedying the effect of lesions in areas of the cerebrum. Previous studies [33, 53] have indicated that there are decreased ReHo and degree centrality values of MFG_R in DON patients. ReHo was used to evaluate local synchronization of adjacent voxels in the whole cerebrum at rest [54], and degree centrality was used to observe the connectivity of cerebrum networks at the voxel level [55]. However, dALFF as an extension of rs-fMRI can better reflect the dynamic electrical activity of the cerebrum in diseases, especially applied in DR [28], consciousness [20], anxiety [56], and depression [57]. One study revealed that DR patients increased dALFF variability in the left parahippocampal gyrus, left cerebellum_8, left cerebellum_9, and right brainstem [28], but we did not found them in DON patients using dALFF method, this difference enlightened us to realize the mechanism of brain dALFF variability in patients with DON or DR may be inconsistent. Visual impairment in DON patients may lead to loss of visual information processing, which could relate to feedback-controlled cerebrum areas in order to repair the ability to turn on endogenous attention. The elevated dALFF of DON patients in the right MFG might be increased activity to deal with attention transforms, which could affect life quality in other aspects, such as anxiety and depression (Figure 5). Correlation analysis with HADS revealed that anxiety and depression scores were negatively correlated with dALFF values of the MFG_R. Therefore, more attention should be paid to emotions of DON patients. These data implied that local synchronization and connectivity in DON patients is abnormal in areas of the cerebrum. In addition, ROC curve showed that MFG_R had high accuracy for diagnosing DON. We conjectured that the MFG_R may be a potential biomarker for DON patients.

It is noteworthy that vision loss can affect the health-related quality of life [58] and easily cause emotional changes in patients, such as anxiety and depression [38, 59] Moreover, low vision correction can significantly improve anxiety and depression indicators in visually impaired patients [59, 60]. Compared with HCs, DON patients had significant vision loss and afferent optic pathway impaired which can affect the normal lives and lead to negative emotions. Huang et al. [61] showed that DR patients exhibited increased dALFF variability in some brain regions that were not observed in our study, it means the increased dALFF values of MFG_R was due to the afferent optic pathway injury in DON patients. In addition, correlation analysis revealed that anxiety and depression scores were strong negative correlation with dALFF values of the MFG_R. According to recent functional imaging studies, the MFG_R are now considered to play an essential role in emotion regulation, and the lesions of this area are prone to show anxiety and depression [62–65]. Some studies [56, 57] demonstrated that patients with generalized anxiety disorder or major depressive disorder exhibited abnormal dALFF variability in widespread regions, but not included MRG_R, this may due to different degree of anxiety and depression or different mechanism between them. Therefore, our study supposed that DON patients may lead to lesions of MRG_R, and then caused the symptoms of anxiety and depression.

We also observed decreased dALFF variability in the PreC_R in patients with DON. Anatomically, the precuneus is located directly in front of a wedge-shaped fold on the medial side of the occipital lobe. The marginal branch of the cingulate sulcus, the medial portion of the parieto-occipital fissure, and the inferior parietal sulcus are the front, rear, and lower edges of the precuneus, respectively [66]. The human cerebrum showed different methods of functional connectivity between disparate cerebrum areas, even at rest [67, 68]. The precuneus, posterior cingulate cortex, medial prefrontal cortex, and bilateral temporoparietal junction formed the default mode network (DMN), which is the network most easily related to rest states, and its activity increases at rest and decreases with task engagement [69, 70]. Across the areas of the DMN, the precuneus is prominent for having a unique role. Two studies [71, 72] have shown that precuneus acts as a key part of the DMN by converting its network connectivity to the left frontal-parietal control network and DMN depending on the situation of the cerebrum. Cavanna et al. [73] demonstrated that the precuneus plays a core role in visuo-spatial imaging, episodic memory retrieval, and self-processing operations. Compared with HCs, DON patients underwent blurred vision and impaired visual image acquisition due to significant worse visual ability, besides, we found that decreased dALFF variability in the PreC_R in patients with DON, which means that the DMN was engaged in the task. We speculated that its abnormal activity may lead to weakening of the ability to regulate rest and work states, and even affect the cerebrum functional connection. Our findings agree with a report by Chen et al. [34]. In this study, they demonstrated abnormal functional connection density in some cerebrum areas of DON patients. As precuneus activation is usually attributed to episodic memory and the visuo-spatial process, the influence of DON deserves special attention when observing cerebrum activity. Moreover, ROC curves in Figure 3 show different signal values of brain areas that exhibited acceptable sensitivity and excellent specificity when the two groups were distinguished, which could lead to the suggestion that the dALFF values of MFG_R and PreC_R may be potential diagnostic markers for DON.

**Figure 6:**
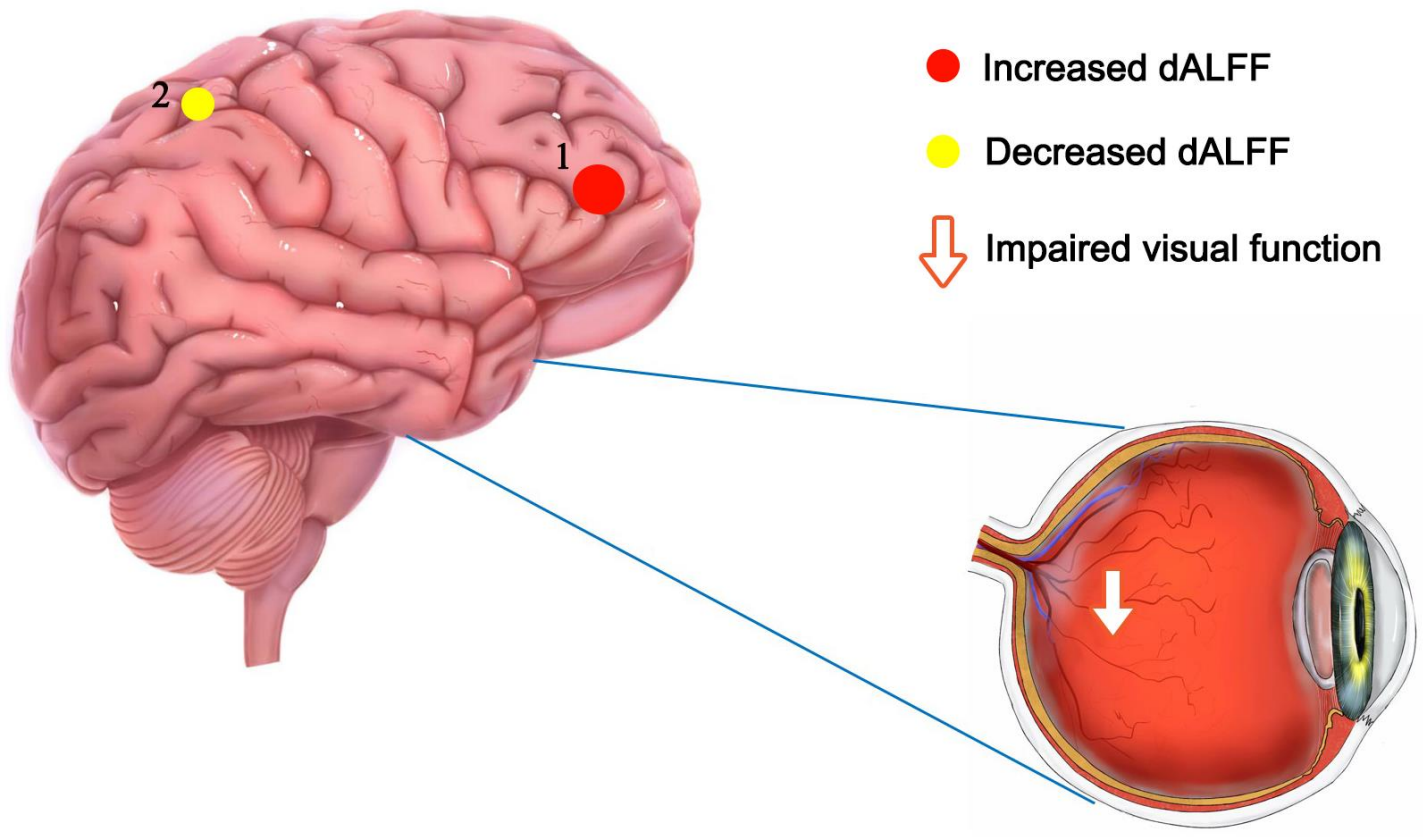
**The mean dALFF values of altered cerebrum regions.** Compared with HCs, the dALFF values of the following region 1 in DON patients was increased: MFG_R (BA 10, t = 4.9595), while the dALFF values of the following region 2 was decreased: PreC_R (t =-3.6307). Abbreviations: dALFF, dynamic amplitude of low-frequency fluctuation; HCs, healthy controls; DON, diabetic optic neuropathy; R, right; MFG, middle frontal gyrus; PreC, precuneus; BA, Brodmann's area.

In this study, we observed cerebrum activity of dALFF in DON patients and healthy controls (Figure 6 and Table 4), which could allow a better understanding of the potential pathophysiological mechanisms of DON patients, and dALFF may be a useful tool for detection and discrimination of the effects of cerebrum activity in DON patients. However, larger sample size studies and longitudinal research should be conducted in the future.

**Table 4:** Alternation of cerebellum regions and its potential impact.

**Cerebellum regions**	**Experimental results**	**Cerebellum functions**	**Anticipate effects**
MFG_R	DON > HCs	Auditory information processing, reorienting of attention	Mental disorders and inattention, including depression and anxiety
PreC_R	DON < HCs	visuo-spatial imaging and episodic memory retrieval processing	Movement and memory impairment, including acute sleep deprivation

Abbreviations: MFG, middle frontal gyrus; R, right; PreC, precuneus; DON, diabetic optic neuropathy; HCs, health control group.

## CONCLUSIONS

In total, patients with DON exhibited increased dALFF of MFG_R implicated in mental disorders and inattention and decreased dALFF of PreC_R implicated in movement and memory impairment. The hyperintensities of MFG_R may be related to the anxiety and depression of DON patients and contributed to distinguishing patients with DON from HCs. This study sheds new insight into the brain dysfunction underlying DON from the perspective of dynamic local brain activity, highlighting the important role of alterations in dALFF variability in understanding the neuropathological mechanisms underscoring DON and potentially informing the diagnosis of this disease.

## MATERIALS AND METHODS

### Participants

Twenty-two DON patients (10 males and 12 females) who had been diagnosed and treated in the Department of Ophthalmology of the First Affiliated Hospital of Nanchang University were enrolled as the DON patient group (DON). The diagnostic criteria of DON was based on Levin et al. [74] as follows: a) a clear history of diabetes; b) optic disc edema (nonspecific congestive edema), or ischemic optic neuropathy, optic disc neovascularization and optic nerve atrophy; c) different degrees of visual impairment, no clinical basis for visual impairment, no typical clinical features on visual field examination, and enlarged physiological blind spots or limited impairment of visual acuity; d) fundus fluorescein angiography revealing early lesions, involving part or all of the optic papilla, or showing blurred, leaky, and low fluorescence; e) exclusion of other diseases that may cause optic disc edema, such as toxic optic neuropathy, hereditary Leber’s optic neuropathy, local ischemic papillary optic lesion, congenital abnormalities of the optic nerve, optic nerve trauma, or systemic lesions; f) disc edema caused by optic neuropathy that could be treated or would recover after approximately 6 months with the recovered disc possibly appearing pale; and g) no other systemic disease, such as high blood pressure.

Twenty-two healthy controls (10 male and 12 female participants) without DM matched for age, gender, handedness, educational level and total intracranial volume were treated as HCs. The inclusion criteria for HCs were as follows: a) no ocular disease history; b) no drug or alcohol abuse history; c) no neurological or psychiatric diseases; d) no MRI contraindications; and e) normal brain parenchyma on MRI.

This study was approved by the medical ethics committee of the First Affiliated Hospital of Nanchang University, which is also compliant with the Declaration of Helsinki. All participants voluntarily participated in the study, understood its methods, purpose, and potential risks, and signed informed consent forms.

### MRI data acquisition

All participants were scanned with a Siemens Trio 3.0T MRI scanner (Trio; Siemens, Munich, Germany) using an 8-channel phased-array head coil. During the 8 min MRI examination, all participants were in a comfortable and noise-free environment and were kept awake. The scanning parameters were performed as follows: repeat time =2,000 ms; echo time =30 ms; field of view =240 mm × 240 mm; flip angle =90°; slice thickness =3 mm with a 1-mm gap; and number of slices = 30. A total of 240 functional images were finally captured.

### rs-fMRI data processing

The rs-fMRI data preprocessing was performed using Data Processing and Analysis for Brain Imaging (DPABI, http://www.rfmri.org/dpabi) [75], which was performed with the following steps: 1) the first 10 time-points were excluded, and slice timing was carried out to correct time differences; 2) realignment for individual-level correction was applied to correct head motion with a Friston-24 model; 3) mean framewise displacement (FD) was used to minimize the potential influences of head motion; 4) several covariates were regressed; 5) the data were normalized to the standard Montreal Neurological Institute (MNI) echo planar imaging (EPI) template at a resolution of 3×3×3 mm^3^; 6) a temporal bandpass filter (0.01–0.08 Hz) was applied; and 7) functional volumes were smoothed with 6-mm full-width at half maximum Gaussian kernel.

### dALFF analysis

The sliding window was applied to quantify the dALFF of each participant using the DynamicBC toolbox (v2.0, http://www.restfmri.net/forum/DynamicBC) [76], which is a significant parameter to evaluate dynamic spontaneous neural activities, and the proper window length played a crucial role in dynamic analysis. Previous studies indicated that the range of appropriate window length was 10–75 TR and step = 1 TR [77, 78]. Therefore, an appropriate sliding window length of 30 TR (step = 1 TR) and five TR (10 s) as the step size was chosen to calculate the dALFF of each participant and maximize the statistical power. Then the ALFF map was computed within each window. To evaluate the temporal variability of dALFF (dALFF variability), we measured the variance of these maps by using the standard deviation. Furthermore, the dALFF variability was transformed into z-scores for statistical analyses.

### Statistical analyses

SPSS version 19.0 software IBM Corporation, Armonk, NY, USA) was applied to analyze the cumulative data. The Chi-square (x^2^) test and independent *t*-test were used to assess the clinical data between two groups. Two-sample t-test was used to compare dALFF values between DON patients and HCs, with age, sex, and mean FD as covariates. and the Gaussian Random-Field (two-tailed, voxel level, P < 0.01; gaussian random field correction, cluster level, P < 0.05) were used to process multiple comparison corrections. P < 0.05 was regarded as statistically significant. In addition, ROC curves were used to compare specific cerebral regions between the two groups.

### Correlation analysis

The HADS was filled out by all patients, and the differences in clinical behavior were based on scores of anxiety and depression. The GraphPad Prism 8 software (GraphPad Inc, San Diego, CA, USA) was applied to analyze Pearson’s correlation, and to evaluate and plot the linear correlation between HADS scores and mean dALFF signal values in the MFG_R.
